# An Energy-Efficient Cluster Head Selection Scheme for Energy-Harvesting Wireless Sensor Networks

**DOI:** 10.3390/s20010187

**Published:** 2019-12-28

**Authors:** Qian Ren, Guangshun Yao

**Affiliations:** 1 School of Computer and Information Engineering, Chuzhou University, Chuzhou 239000, China; renqian99@chzu.edu.cn; 2 School of Computer Science and Engineering, Southeast University, Nanjing 211189, China

**Keywords:** energy-harvesting wireless sensor networks, cluster head selection, scheduling node, routing

## Abstract

Concerning the large amount of energy consumption during the cluster head selection stage and the unequal harvested energy among nodes in energy-harvesting wireless sensor networks (EH-WSNs), an energy- efficient cluster head selection scheme called EECHS is proposed in this paper. The scheme divides all nodes from one cluster into three types: cluster head (CH), cluster member (CM), and scheduling node (SN). The SN is designed to monitor and store real-time information about the residual energy of all nodes, including CMs and the CH, in the same cluster. In the CH selection stage, the SN specifies a corresponding CM as the new CH according to the monitored results, thereby reducing the energy consumption caused by CH selection. In this way, the task of CH selection is migrated from CHs to SNs and, thus, the CHs can preserve more energy for data forwarding. Moreover, the EECHS adjusts the transmission radius of some nodes dynamically to prevent these nodes from discarding the harvested energy if their batteries are fully charged. A series of experiments were conducted to verify the effectiveness of the proposed EECHS, and the results demonstrate that EECHS can provide an efficient CH selection scheme for EH-WSNs and is able to use the harvested energy more efficiently than corresponding competitors.

## 1. Introduction

Recently, wireless sensor networks (WSNs) have been widely used in many applications, such as environmental monitoring, biomedical health monitoring, and target tracking. Cluster-based routing is an energy-efficient scheme for WSNs to prolong the network lifetime [1,2]. The clustered WSNs are typically composed of a certain number of clusters and a sink node. Each cluster usually contains some cluster member (CM) nodes and a cluster head (CH) node. The CHs usually play an important role in cluster-based WSNs as they are responsible for receiving and processing data from CMs in the same cluster with this CH and for then forwarding the collected data to the sink, either directly or via multi-hop routing by other CHs [3]. In past decades, many cluster-based routing algorithms, such as low-energy adaptive clustering hierarchy (LEACH) [4] and energy-efficient unequal clustering (EEUC) [5], have been proposed for traditional WSNs. Many CH selection and energy-efficient routing schemes have been introduced in these cluster-based routing algorithms. However, the energy consumed for CH selection has not attracted much attention. As the sensor nodes in traditional WSNs are usually powered by batteries and the exhaustion of batteries is inevitable [6], designing an energy-efficient CH selection scheme is valuable for cluster-based routing to prolong the lifetime of WSNs.

**Figure 1:**
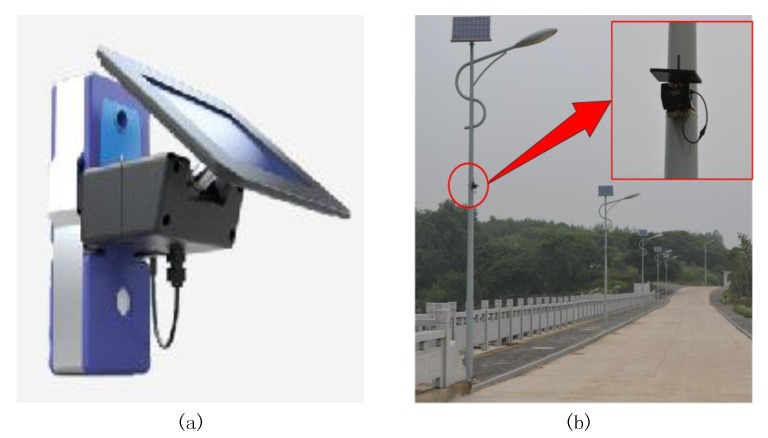
(**a**) A wireless sensor node with a solar panel and a harvested battery. (**b**) A sensor node as shown in Figure 1a is installed on a utility pole to monitor environmental information.

When each sensor node is equipped with an energy-harvesting component as shown in Figure 1 (Figure 1a shows a wireless sensor node with a solar panel and a harvested battery while Figure 1b exhibits that the sensor node is installed on a utility pole to monitor environmental information), the design becomes even more difficult as the node can harvest energy from external sources (e.g., solar, thermal, vibration, and Radio Frequency energy), and the perished sensor nodes in energy-harvesting wireless sensor networks (EH-WSNs) can revive again after harvesting [3,7,8]. Although nodes can harvest energy from the environment, different nodes in EH-WSNs usually have different energy-harvesting efficiencies [3]. For example, if sensor nodes such as some that are used in precision agriculture for environmental monitoring only harvest energy by solar panels, the nodes in shady areas will harvest less energy than the nodes in sunny areas [9]. Moreover, the rechargeable battery capacity is usually limited, and the harvested energy is discarded after the battery has been fully charged [10]. In this situation, how to select reasonable CHs and to set the working scheme of both CHs and CMs for using the harvested energy efficiently becomes a very important issue.

In this paper, an energy- efficient cluster head selection scheme called EECHS is proposed for EH-WSNs. In EECHS, one sensor node in each cluster is selected and defined as the scheduling node (SN). The main task of the SN in the data collection stage is to monitor the energy of all CHs and CMs from the same cluster with this SN in real time. Thus, the nodes in each cluster are divided into three types in EECHS: CH, CM, and SN. In the CH selection stage, the main task of the SN is to specify one node as the new CH according to the monitored result for the next round. By this scheme, the task of CH selection is migrated from CHs to SNs, and thus, the CHs can preserve more energy for data forwarding. Furthermore, as the new CHs are appointed by SNs directly, the transmitted message for CH selection is reduced and less message and energy is required for CH selection. Moreover, the sensor nodes—including CH and CMs—in EECHS dynamically adjust their working model to transmit data to the sink by either multi-hop or one hop directly according to their residual energy and the capacity of the rechargeable battery. In this way, the harvested energy is used more efficiently. The main contributions of this paper are as follows:

- An SN is designed for each cluster to monitor and store real-time information about residual energy for all CMs and the CH in the same cluster. During the CH selection stage, the designed SN specifies a corresponding CM as the new CH according to the monitoring results and thus decreases the workload of the CH to preserve more energy for data forwarding. It also reduces the consumed energy for CH selection.
- The transmission radius of nodes—including CH and CMs—is adjusted dynamically in EECHS for efficient utilization of the harvested energy.
- Extensive experiments were conducted, and the experimental results verify the effectiveness of EECHS in selecting high-quality CHs in a low-energy-consumption scheme that utilizes the harvested energy efficiently.

The rest of the paper is organized as follows. The related work is reviewed in Section 2. Section 3 introduces relevant models, including the network and energy consumption model used in this paper. The proposed algorithm is depicted in Section 4. Section 5 presents the performance evaluation of the proposed algorithm, followed by conclusions and future work in Section 6.

## 2. Related Work

The selection of some nodes as CHs is an important part of cluster-based routing algorithms. In past decades, many CH selection schemes have been designed and involved in different cluster-based routing protocols. In Reference [5], Chen et al. proposed energy-efficient unequal cluster-based routing for WSNs. It selected the CH of a cluster based on the residual energy of all nodes in the same cluster. Gupta and Pandey [11] proposed a different energy-aware distributed unequal clustering method for WSNs. The CH in Reference [11] is selected based on the current residual energy of one node and the current average residual energy of this node’s neighbors. In contrast to Reference [11], Razaque et al. [12] selected CHs based on the residual energy of the sensor node and the distance from the node to the base station. Darabkh et al. [13] proposed two clustering algorithms for target tracking in WSNs. In order to satisfy the requirement of target tracking, the CH in Reference [13] is selected based on the node’s residual energy, the distance between the detecting sensor nodes and the sink node, and the distance between the target and detecting sensor node. In contrast to the above work, Lin et al. [14] proposed an energy-efficient clustering strategy for WSNs that selects a node as CH in a precalculated central area of each cluster. Some other researchers also proposed different efficient CH selection strategies for WSNs from different perspectives. Shalini and Vasudevan [15] proposed an enhanced dynamic cluster head selection method to decrease the overlapping coverage and unbalanced energy consumption inside the cluster communication caused by unreasonable cluster head selection. For a similar problem, Priyadarshi et al. [16] selected two CHs for each cluster. Similarly, Naranjo et al. [17] adopted two heterogeneous nodes for energy-limited heterogeneous fog-supported WSNs. The CHs are selected based on predefined energy thresholds and weighted probabilities for both the normal and advanced nodes. Some researchers have also proposed some CH selection algorithms based on nature-inspired approaches, such as the artificial bee colony [18], firefly [19], particle swarm optimization [20], and chemical reaction optimization algorithms [21,22]. However, all these algorithms are designed for traditional WSNs powered by non-rechargeable batteries and cannot be used in EH-WSNs directly. Furthermore, selecting new CHs is usually triggered and completed by current CHs in the above algorithms (except for the nature-inspired approaches), and the energy consumed for selecting new CHs is not considered. Similar to our work, Zhang et al. [23] proposed a cluster-based routing algorithm called BEN, taking the energy consumed for completing new CHs into consideration. It also assigned a sensor node as the SN for each cluster to monitor the residual energy of each CM and CH in the same cluster. However, similar to the above algorithms, it was also not designed for EH-WSNs and cannot be used in EH-WSNs directly.

Recently, Zhang et al. [24] selected some energy-harvesting nodes as relay nodes for cluster-based routing. The data relay task undertaken by CHs in other algorithms is completed by the relay node in their work. After calculating the best position of CHs, Zhang et al. [25] deployed some energy-harvesting nodes as CHs to prolong the network lifetime, which is similar to Reference [24]. However, only the relay node is equipped with an energy-harvesting component. Thus, the used WSNs models in both References [24] and [25] are different from the EH-WSNs model considered in this paper, where each node has an energy-harvesting component. Moreover, the limited battery capacity is not considered in Reference [24], and the uneven harvested energy among nodes is not considered in Reference [25], even though this is very common in practice.

For EH-WSNs, researchers have also proposed some cluster-based routing algorithms and CH selection schemes. Peng et al. [26] proposed a distributive energy-neutral clustering (ENC) protocol to group the EH-WSNs into several clusters with the goal of providing perpetual network operation. ENC selects several nodes to comprise the cluster head group (CHG) and the selected CHG plays the role of the CH. However, the data from the area of the CHG is missed. Yukun et al. [27] proposed an improved clustering routing algorithm called CRAS for EH-WSNs from a different perspective. After considering the current residual energy and harvested energy, CRAS selects CHs using a scheme similar to the one proposed in LEACH [4]. Moreover, CRAS also considers the uneven harvested energy among nodes when designing the routing algorithm. However, the capacity limitation of the rechargeable battery is not considered, and thus, a great deal of harvested energy is discarded in CRAS. To the best of our knowledge, the problem considered in this paper has not yet been studied, even though it occurs widely in practice.

## 3. Network and Energy Consumption Model for EH-WSNS

### 3.1. Network Model for EH-WSNs

The EH-WSNs considered in this paper can be used for many applications, such as environmental monitoring for precision agriculture. The considered EH-WSNs contain *N* wireless sensor nodes and a sink node. Each sensor node is powered by a rechargeable battery with limited capacity and is also equipped with a photovoltaic panel that can harvest solar energy and store the harvested energy in the rechargeable battery. The EH-WSNs are divided into multiple unequal clusters, and each cluster contains some CMs and a CH. The main tasks of the CMs in a cluster are to sense and transmit the collected data to the CH located in the same cluster as them. The main task of the CH is to receive and transmit the data from CMs in the same cluster and to relay the data from other clusters to the sink by multi-hop or one-hop routing [3].

We suppose that the considered EH-WSNs  has the following characteristics: 1) It is a static network, where all nodes, including the sink node, cannot move after deployment. 2) There are *N* nodes in the monitored area and one sink located outside of the monitored area. Each sensor node has a unique ID i(1≤i≤N) and has the capability of data fusion. The distance between node *i* and the sink is denoted as $d_{i}$. The maximum and minimum distances between one sensor node in the network and the sink are denoted as $d_{max}$ and $d_{min}$, respectively. 3) The rechargeable battery capacities of all nodes are identical and denoted as $E_{cap}$. The initial energies of all nodes are identical and denoted as $E_{ini}$. The residual energy of node *i* is expressed as $E_{i}$ after a period of work. 4) The transmission range of the sink can cover the entire deployment area, and the wireless transmission power of each node can be adjusted based on the distance between the receiver and itself. 5) All nodes are kept in listening state before establishing the clusters. All the nodes in a cluster are divided into three types: CH, CM, and SN. The type of all nodes in a cluster is set as normal at the beginning of the first round—that is, $kind_{i}=normal\left(1\leq i\leq N\right)$.

### 3.2. Energy Consumption Model

If the distance between two sensor nodes is *d*, the energy consumed by one sensor node sending $k_{1}$ bit of data to another node is defined as follows [3,5,23]:

(1) $$E_{TX}\left(k_{1},d\right)=k_{1}\times \left\{\begin{array}{c} E_{tx}+\varepsilon_{fs}\times d^{2}\;d<d_{0}\\ E_{tx}+\varepsilon_{mf}\times d^{4}\;d\geq d_{0} \end{array}\right.,$$

The energy consumed for the other sensor node receiving $k_{1}$ bit of data is defined as follows:

(2) $$E_{RX}\left(k_{1},d\right)=k_{1}\times E_{rx}.$$

In general,

(3) $$E_{tx}=E_{rx}=E_{ele},$$

where $E_{ele}$ represents the circuit energy loss and usually depends on factors such as the digital coding and modulation. $\varepsilon d^{n}$ represents the amplifier energy and is usually decided by the transmission distance and the acceptable bit-error rate. $\varepsilon_{fs}$ and $\varepsilon_{mf}$ are the propagation loss coefficients. The value of *n* is decided by the transmission distance. In general, a predefined threshold $d_{0}=\sqrt{\varepsilon_{fs}/\varepsilon_{mf}}$ is used to decide the value of *n*. If *d* is no less than $d_{0}$, n=4; otherwise, n=2. Let $d_{ij}$ denote the distance between nodes *i* and *j*. Then, the energy consumed for node *i* transmitting $k_{1}$ bit of data to node *j* can be denoted as follows:

(4) $$E_{TX}\left(k_{1},d\right)=k_{1}\times \left\{\begin{array}{c} E_{ele}+\varepsilon_{fs}\times d_{ij}^{2}\;d_{ij}<d_{0}\\ E_{ele}+\varepsilon_{mf}\times d_{ij}^{4}\;d_{ij}\geq d_{0} \end{array}\right..$$

Thus, the residual energy of node *i* is updated after transmitting $k_{1}$ bit of data as follows:

(5) $$E_{i}=E_{i}-E_{TX}\left(k_{1},d\right).$$

The consumed energy used for node *j* receiving $k_{1}$ bit of data is

(6) $$E_{RX}\left(k_{1},d\right)=k_{1}\times E_{ele}.$$

During the working process, the CH node needs to collect the data sent by the CM nodes from the same cluster. At the same time, it is also required to relay the data from other clusters. Thus, the energy consumption of one CH node *j* for completing the above tasks is

(7) $$E_{RX}^{CH}\left(k_{1},d\right)=\left(k_{2}+k_{3}\right)\times E_{RX}\left(k_{1}\right)+E_{TX}\left(k_{1},d\right),$$

where $k_{2}$ is the number of CMs in the same cluster of this CH node and $k_{3}$ is the number of clusters that need to relay data to the sink through node *j*. After completing the above tasks, the residual energy of the CH node *j* is updated as follows:

(8) $$E_{j}=E_{j}-E_{RX}^{CH}\left(k_{1},d\right).$$

## 4. Algorithm Implementation

The working process of the EH-WSNs in this paper is divided into two stages: the cluster establishment stage and the data collection stage. The main task of the first stage is to divide the whole monitored area into multiple unequal clusters and to select an SN as well as a CH for each cluster. The data collection stage, which adopts a round-based scheme as in LEACH [4], is divided into the data transmission stage and the CH selection stage. In the following sections, we elaborate on the details of these substages.

### 4.1. The Cluster Establishment Stage

This stage is the period needed for initial clustering before the EH-WSNs start working. The main task of this stage is to divide the whole area of EH-WSNs into multiple unequal clusters and to select an SN and a CH for each cluster.

After all nodes are deployed, the sink sends a message $Partion\_Cluster(d_{max},d_{min})$ to all nodes for clustering. After receiving $Partion\_Cluster(d_{max},d_{min})$, each sensor node calculates its distance to the sink (i.e., $d_{i}$ for node *i*) according to the strength of the received signal. Then, node *i* sends a message $Cluster(i,d_{i},E_{i},R_{i}^{c})$ for clustering, where $R_{i}^{c}$ is the competitive radius for node *i* and can be denoted as follows:

(9) $$R_{i}^{c}=\left(1-\alpha \frac{d_{max}-d_{i}}{d_{max}-d_{min}}\right)R^{C},$$

where α is a constant coefficient between 0 and 1 and $R^{C}$ is the predefined maximum competition range of all nodes.

**Figure 2:**
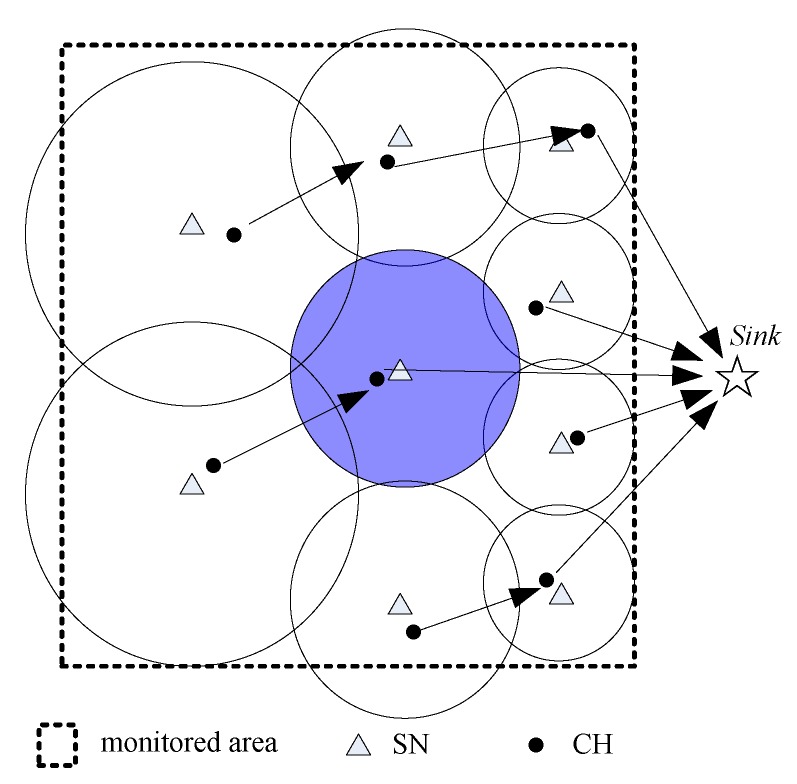
The energy-harvesting wireless sensor network (EH-WSN) model used in this paper. CH: cluster head; SN: scheduling node.

Then, the method proposed in Reference [5] is adopted to divide the whole monitored area into multiple unequal clusters as shown in Figure 2. Moreover, the clusters closer to the sink are smaller than those farther from the sink. Thus, the CHs closer to the sink can consume less energy for the intra-cluster data forwarding and can preserve some energy for the inter-cluster data forwarding. In addition to dividing the monitored area into multiple unequal clusters, the method proposed in Reference [5] is also used to select a sensor node in a cluster with the maximum residual energy as the CH for this cluster. Then, the CH allocates a time spot for all CMs in the same cluster. More details about unequal clustering and CH selection are given in Reference [5]. Note that each sensor can determine the deployed location of other nodes in the same cluster after the cluster establishment stage by some localization algorithms, which is beyond the scope of this paper.

Then, each cluster needs to select a node as the SN. In the following section, we take one cluster as an example to explain the corresponding working process of EECHS. Let *i*, *j*, *n*, and *ℵ* denote the ID of SN, CH, one CM node in this cluster, and the number of CMs in this cluster, respectively. After clustering, each node in this cluster can get the location information of other nodes in the same cluster. So, the node *i* at the center of this cluster is selected as the SN. If no such node exists; the node with the shortest distance from the center of this cluster is selected. Then, the SN node *i* extracts the information about the residual energy of all other nodes in the same cluster from $Cluster(n,d_{n},E_{n},R_{n}^{c})$. After that, the SN *i* constructs an energy table (denoted as EneTab) for this cluster, and the structure of EneTab is expressed as {⋯, node ID *n*, node type $kind_{n}$, residual energy $E_{n}$,⋯}.

### 4.2. Data Collection Stage

This stage is used for collecting the monitored data after the cluster establishment stage and is the main stage of EH-WSNs. The round-based scheme similar to the one used in LEACH is adopted in this stage, and each round is divided into two substages: the data transmission stage and the CH selection stage. During the data transmission stage, the CMs collect the sensing data and periodically send it to the CH node of the cluster. The CH node collects the transmitted data from all CMs in the same cluster and the relayed data from other CHs and then sends these data to the sink through multi-hop or one hop directly. After the CHs periodically collect data *T* (T>1) times, the EH-WSNs trigger the CH selection stage to select other suitable nodes as new CHs for the next round. In this way, the task of CH selection is migrated from CHs to SNs. Thus, the CHs can preserve more energy for intra-cluster and inter-cluster data forwarding. Furthermore, the new CH for the next round is specified by SN directly and thus less messaging and energy is required for CH selection.

**Figure 3:**
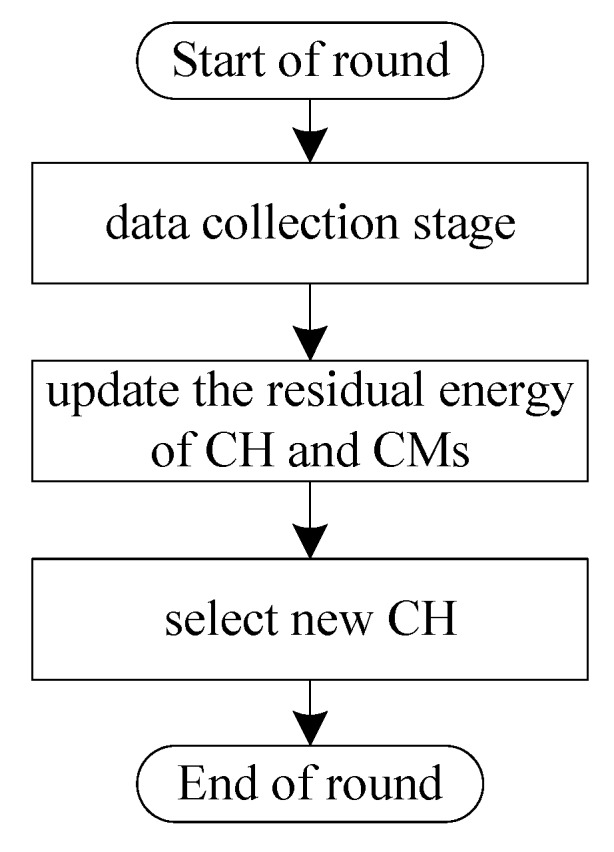
The flowchart for the working process of an SN in a round. CM: cluster member.

The flowchart for the working process of SN is shown in Figure 3. Let *i*, *j*, and *n* denote the ID of the SN, CH, and one CM node of this cluster, respectively. Let $j^{\prime }$ and *ℵ* denote the ID of the new selected CH after the CH selection and the number of CMs in this cluster, respectively. Note that the residual energy of each node is updated in two ways. It is decreased by Equation (5) or Equation (8) after sending a message on one hand and is increased after harvesting energy from the environment on the other hand. The main pseudo code of the SN node in EECHS is shown in Algorithm 1.

During the data transmission stage, the CM node *n* only needs to wake up in its own time spot to collect data and to send it to the CH node, while the CH node *j* needs to collect and transmit the data sent by the CMs in the same cluster as well as the data sent by other CHs. When only considering the energy consumption by receiving and sending messages, the residual energy of CMs decreases according to Equation (5) while that of the CH decreases according to Equation (8). Thus, the SN node updates the corresponding residual energy of CH and CM according to Equation (8) and Equation (5), respectively (lines 3–10). Note that the CH will broadcast a request message for CH selection if its residual energy is less than or equal to $\beta \times E_{ini}$ (β is a constant coefficient between 0 and 1) during the data collection stage, as introduced in the following process. Under this situation, the corresponding SN will receive the request message and go to next process to confirm the correctness of the residual energy values in EneTab. However, in addition to consuming energy, each sensor node in EH-WSNs can harvest energy from the environment and the nodes may harvest different amounts of energy from the environment in a given round if some of them are located in sunny areas while others are in shady areas. Therefore, the SN node broadcasts a message Energy_Con(i,EneTab) for residual energy confirmation. Then, the SN node changes to the listening state to await the answer message $Energy\_Answer(j,E_{real\_j})$ from the CH and $Energy\_Answer(n,E_{real\_n})$ from CMs. If the SN node receives $Energy\_Answer(n,E_{real\_n})$ (or $Energy\_Answer(j,E_{real\_j})$), it updates the corresponding information about residual energy in EneTab (lines 12–18). Then, the SN node selects the node $j^{\prime }$ with the maximal residual energy from the EneTab as the new CH and then broadcasts a message $CH\_Chang(j,j^{\prime })$ to change the CH from node *j* to $j^{\prime }$ for the next round. In this way, the task for selecting CHs is allocated to SNs rather than CHs. Thus, the workload of CHs is reduced and they can preserve more energy for data forwarding. Moreover, the new CHs are specified by SNs directly and thus less messaging and energy is required for CH selection, which further decreases the energy consumption of the EH-WSNs. Then, the SN node changes into the listening state to await the CH win message $CH\_Win(j,j^{\prime })$ from the new CH to confirm that the CH has been successfully replaced (lines 20–26).
**Algorithm 1:** SNWorking
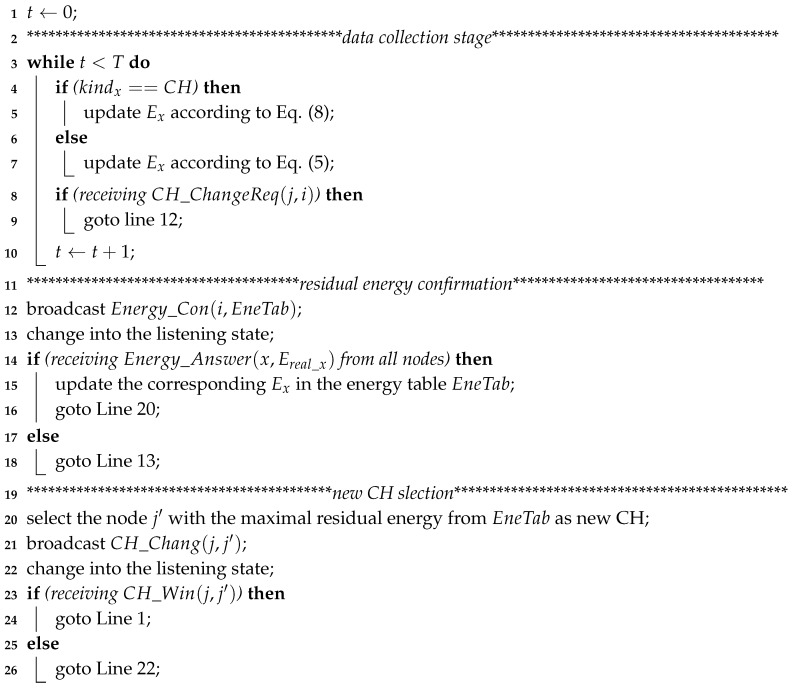


Note that the distance between the CH and CMs in the same cluster is not considered when selecting the CH, as all nodes are within one hop of any node in the same cluster.

We now analyze the complexity of Algorithm 1. There are *ℵ* nodes in a cluster, and each node needs to send the collected data to the CH in the same cluster or sink *T* times during the data transmission stage (lines 3–10). Therefore, the time complexity of this substage is O(ℵ×T). Then, the CH selection substage is triggered and comprises two processes: residual energy confirmation and new CH selection. During the first process, the SN sends a message and then waits to receive the corresponding answer to confirm the residual energy of both the CH and CMs in the same cluster (lines 12–18). The time complexity of this process is O(ℵ). During the last process, the SN specifies a node with the maximal residual energy as the new CH for the next round according to the monitored result in the previous process. Then, the SN broadcasts a message about the CH change to all nodes and waits for the corresponding answers (lines 20–26). Therefore, the time complexity of the new CH selection process is O(ℵ). As a result, the overall time complexity of Algorithm 1 is O(ℵ×T+ℵ+ℵ)=O(ℵ×T).

**Figure 4:**
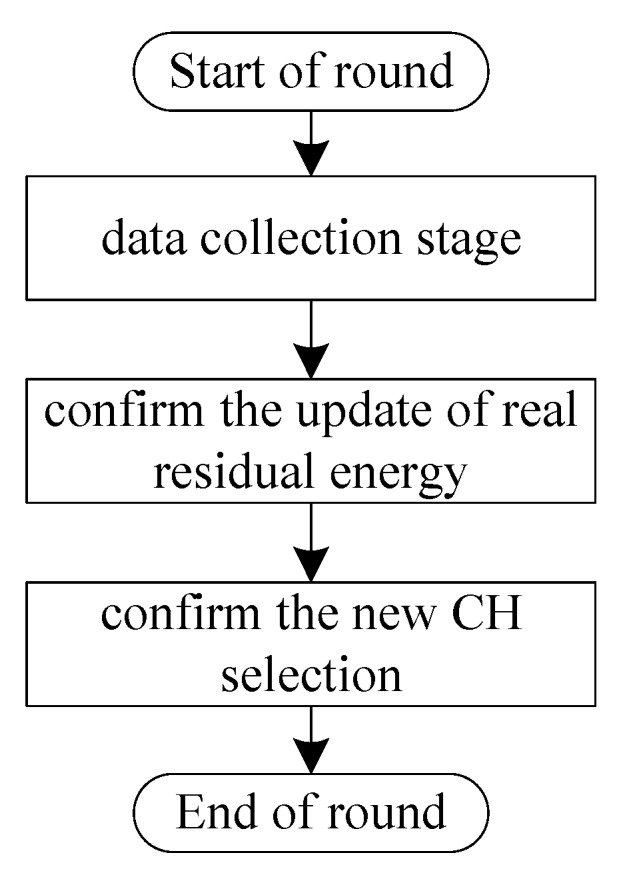
The flowchart for the working process of sensor nodes (including the CH and CMs) in one round.

The flowchart for the working process of sensor nodes (including the CH and CMs) is shown in Figure 4, and the main pseudo code is shown in Algorithm 2.

If the rechargeable battery of a sensor node is already saturated, then the harvested energy is discarded. To take full advantage of the harvested energy, if the rechargeable battery of the CH/CM node has reached its maximum charge capacity $E_{cap}$ in one round, it adjusts its data transmission radius and sends data to the sink directly. Otherwise, it sends data to the sink by multiple hops (lines 3–18). The cluster marked with purple in Figure 2 is an example of a cluster sending a packet to the sink directly. For the CH node during this stage, if its residual energy has been reduced to a predefined threshold (i.e., $\beta \times E_{ini}$), it broadcasts a request message CH_ChangeReq(n,i) for CH selection. After each round, the EH-WSNs run into the CH selection stage and node *x* (CH or CM) changes into the listening state. When receiving the residual energy confirmation message Energy_Con(i,EneTab) from SN, node *x* compares the real residual energy $E_{real\_x}$ with $E_{x}$ extracted from EneTab and broadcasts the energy comparison answer message $Energy\_Answer(x,E_{real\_x})$ (lines 20–24). After the energy confirmation, all nodes change into the listening state. When receiving the CH change message $CH\_Chang(j,j^{\prime })$ from the SN, nodes *j* and $j^{\prime }$ first change their types to CM and CH, respectively. Node *j* also gets the time spot of node $j^{\prime }$, and node $j^{\prime }$ broadcasts the CH win message $CH\_Win(j,j^{\prime })$. For other nodes, a new round is started after they receive the CH win message $CH\_Win(j,j^{\prime })$ and update the CH from node *j* to $j^{\prime }$ (lines 26–42).

Now, we analyze the time complexity of Algorithm 2. As the working process of sensor nodes is similar to the process of the SN in Algorithm 1, the time complexity of Algorithm 2 is O(ℵ×T).

Note that only two messages ($CH\_Chang(j,j^{\prime })$ and $CH\_Win(j,j^{\prime })$) need to be sent by each node during the process for new CH selection in one cluster. This greatly reduces the amount of transmitted packets in the process of CH selection and thus decreases the energy consumed for selecting the new CH. Furthermore, in each data transmission stage, each CM only needs to wake up in its own time slot and remains in a low-power listening state in other time slots to save energy. As for the SN node, it only needs to monitor the residual energy of other CMs and the CH in the same cluster and selects a CM as the CH for the next round. It does not need to sense the data periodically from the environment. Therefore compared with CMs and CH, it consumes less energy. Furthermore, the SN also can harvest energy from the environment by its photovoltaic panel. Therefore, it can hold enough energy for its sustained operation.
**Algorithm 2:** SeneorNodeWorking
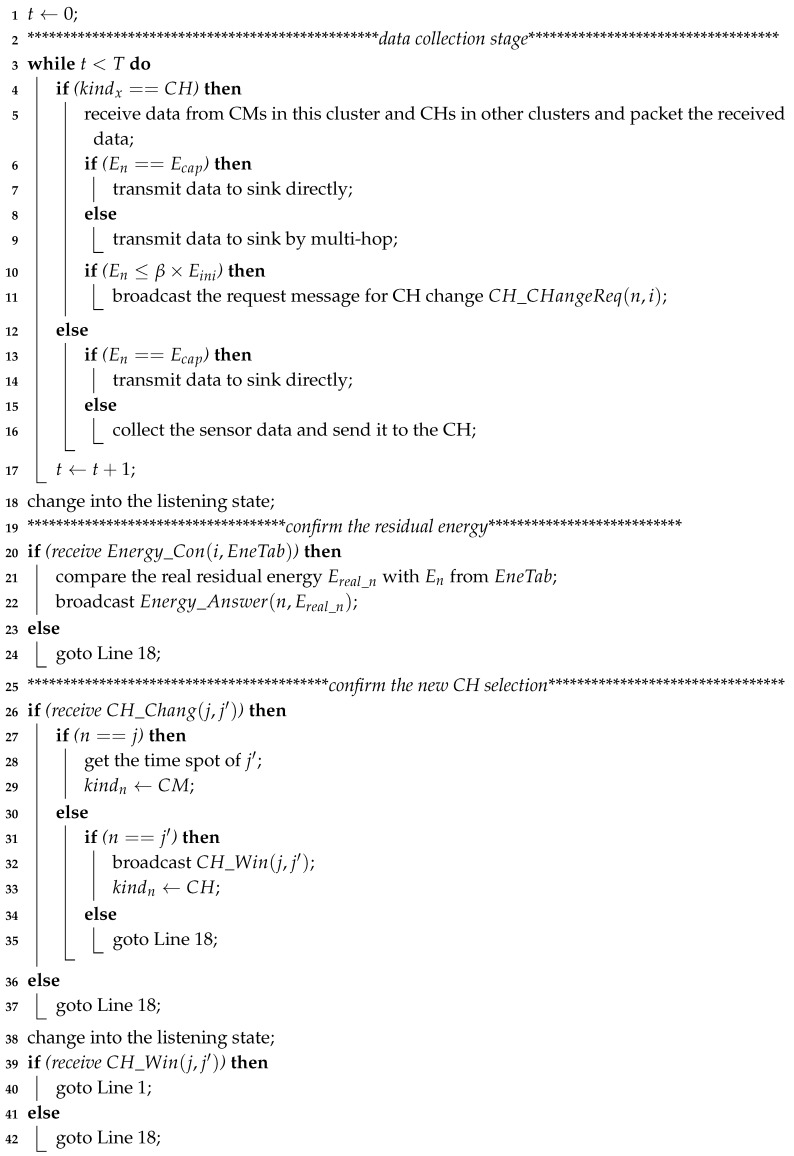


## 5. Performance Evaluation

### 5.1. Simulation Setup

To evaluate the performance of the proposed EECHS, NS-3 was selected as the simulation platform because it produces results that are highly similar to real environments. The simulation environment was configured as follows. For the simulation, we deployed 300 sensor nodes in a two-dimensional area (500 m × 500 m) and one sink node at (505 m, 250 m). For simplicity, we assumed that all sensor nodes were deployed in the first quadrant of the coordinate region. Each sensor was equipped with a harvested battery, of which the maximal capacity was 100 J, and a solar panel with a dimension 10 mm × 10 mm. The updated National Solar Radiation Database statistical summaries [28], which hold solar and meteorological data for 1454 locations in the United States, was used as the solar power harvesting characteristic during the simulation. We also selected 20% of nodes randomly to be deployed in shady areas and assumed that the energy-harvesting rate of nodes in shady areas was 30% of the harvesting rate in sunny areas. All the parameters for simulations are shown in Table 1.

Although many CH selection algorithms have been proposed in the past several decades, they were designed for traditional WSNs and cannot be used in EH-WSNs directly. Thus, we compared the proposed EECHS with CRAS [27], as CRAS is a cluster-based routing algorithm for EH-WSNs and considers the uneven harvested energy among nodes. Besides CRAS, we also compared EECHS with BEN [23], although BEN is designed for traditional WSNs. This is because BEN assigns one sensor node as the SN for each cluster, which is similar to the scheme used in EECHS. When using BEN in EH-WSNs, the proposed CH selection scheme is performed. However, some characteristics in EH-WSNs, such as the uneven harvested energy among nodes, are not considered.

The following metrics were used to evaluate the performance of all these algorithms:

(1) CH quality was defined as the ratio between the residual energy of a CH and the average residual energy of CMs in the same cluster with this CH, reflecting the quality of this CH, as the CH plays an important role in cluster-based routing as mentioned in References [1,2,3]. Ten CHs were selected randomly from each compared algorithm. For each selected CH, we calculated the corresponding quality of the CH from ten randomly selected rounds and used the average result to reflect this metric.

(2) CH quality of CH after one round was defined as the ratio between the residual energy of a CH after a round and the average residual energy of CMs in the same cluster, reflecting the quality of the CH after a round [3]. In EH-WSNs, nodes can harvest energy from the environment and the residual energy of nodes is dynamically changed. After the CH completes its task in a given round, it should keep working as a CM or CH in the next round. Therefore, each CH should use energy efficiently, especially the harvested energy in EH-WSNs, and save enough energy for the next round.

(3) Ratio of packet loss of these algorithms was defined as the ratio between the number of collected packets sent by CMs and the number of received packets from the sink, reflecting the reliability of data transmission.

(4) Average delay of packet delivery was defined as the average delay taken by all packets delivered from their initial sensor nodes to the sink, reflecting the timeliness of information transmission.

(5) Average utilization of available energy from the environment was defined as the ratio of harvested energy from all sensors to the available energy from the environment to all sensors during each time quantum, reflecting the efficiency of each algorithm in using environmental energy.

We ran the compared algorithms 10 times and compared the average results of all simulations for each metric. For the first two metrics, ten CHs were selected randomly from each compared algorithm at each time, and every metric of each selected CH was calculated for ten randomly selected rounds. For the third and fourth metrics, ten rounds were selected randomly from each compared algorithm at each time. For the last metric, the time quanta were selected randomly during simulation and each time quantum contained five rounds.

### 5.2. Evaluation of Experimental Results

**Figure 5:**
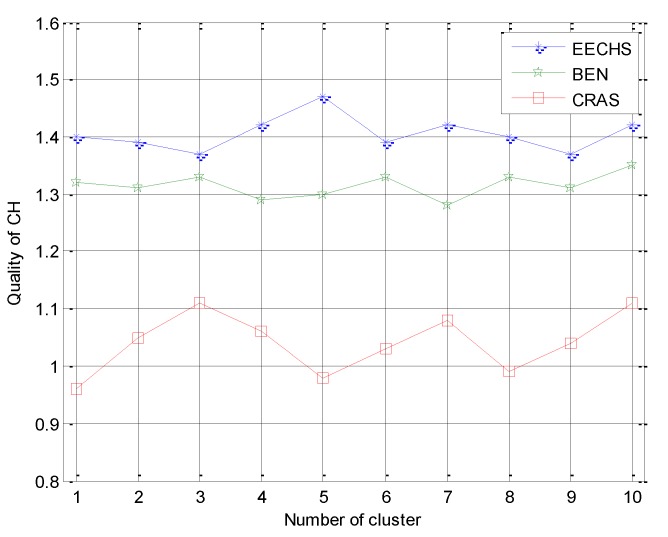
Experimental results for CH quality.

First, we compared the quality of CH, and the average compared results of this metric are shown in Figure 5, which indicates that the quality of each selected CH from both the proposed EECHS and BEN was greater than one, while parts of this metric from CRAS were less than one. This means that the CHs selected in EECRH and BEN had more residual energy than the CHs selected in CRAS. This can be explained as follows. The SN of each cluster in the proposed EECHS holds an energy table EneTab for this cluster. The residual energy of all CMs and the CH are stored and updated in real-time. After each round, the SN selects the node with the maximum residual energy as the new CH for the next round. For BEN, the residual energy of each node is also updated. However, it is not designed for EH-WSNs and the energy harvested from the environment is ignored. In CRAS, although the residual energy is considered as an element to choose the new CHs for each round, similar to LEACH, a random number is selected to choose new CHs and this cannot guarantee that the selected CH has the maximum residual energy in a given cluster.

**Figure 6:**
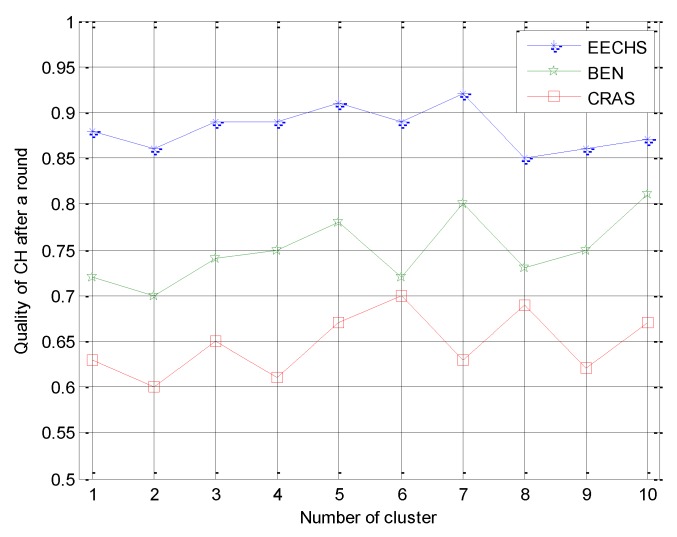
Experimental results for incremental energy of the CHs during the energy-harvesting process.

Second, we focused on the CH quality after one round of these algorithms. The compared results are shown in Figure 6, which shows that the proposed EECHS provided a higher CH quality after one round than the other two competitors, and CRAS had the worst CH quality after one round. BEN always selects a multi-hop path to transmit data without considering the residual energy of CHs. The other two algorithms transmit data to the sink directly or by multiple hops. CRAS transmits data to the sink directly if the harvested energy is greater than the energy consumed for transmission, even when the CHs have less residual energy. As a result, more energy is consumed for CHs transmitting data to the sink directly and less energy is left in the CHs after a round. This reduced amount of energy left in CHs in CRAS reduces the CH quality after one round. The proposed EECHS in this paper transmits data to the sink directly only when the capacity of the rechargeable battery is equal to its limitation $E_{cap}$. Otherwise, it selects a multi-hop route. In this way, the harvested energy is used efficiently and more energy is saved for CHs after a given round.

**Figure 7:**
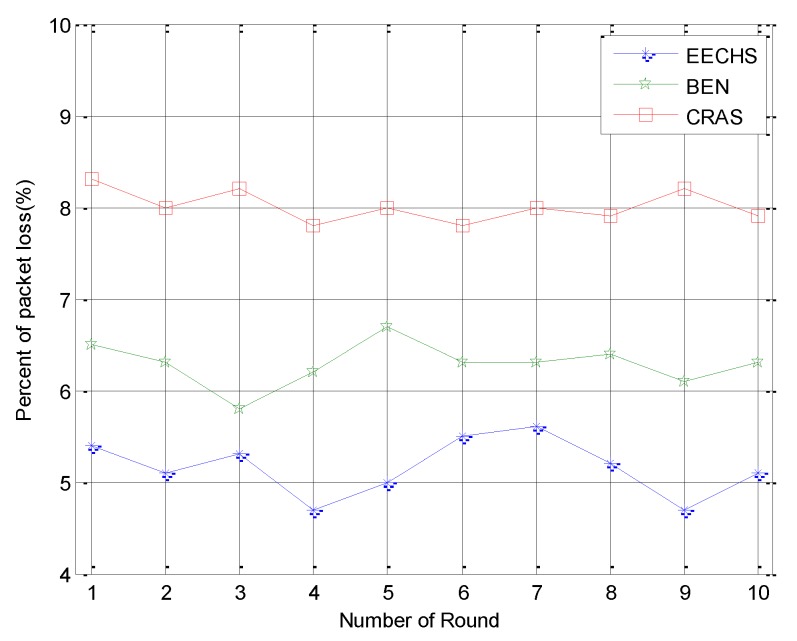
Experimental results for the packet loss ratio.

Next, we compared the ratio of packet loss among these algorithms and the results are given in Figure 7. This figure indicates that the proposed EECHS lost less data than the other algorithms. In general, the ratio of packet loss rate was proportional to both the distance between the sending and receiving nodes as well as the residual energy of the sending and receiving nodes. From the above comparisons, we can conclude that the proposed EECHS selected nodes with more residual energy than the CHs for transmitting data. There was also a high probability of packet loss caused by the long distance between CHs and the sink when sending data to the sink directly. Transmitting directly to the sink was adopted more frequently in CRAS than in EECHS. Therefore, CRAS lost more packets than EECHS. Secondly, after changing its type from CH to CM after a round, a node needs enough energy to complete the task as a CM. However, CHs could not save enough energy for the tasks of a CM after one round, as shown in Figure 6. As a result, more packets were lost in both CRAS and BEN, increasing their packet loss ratios.

**Figure 8:**
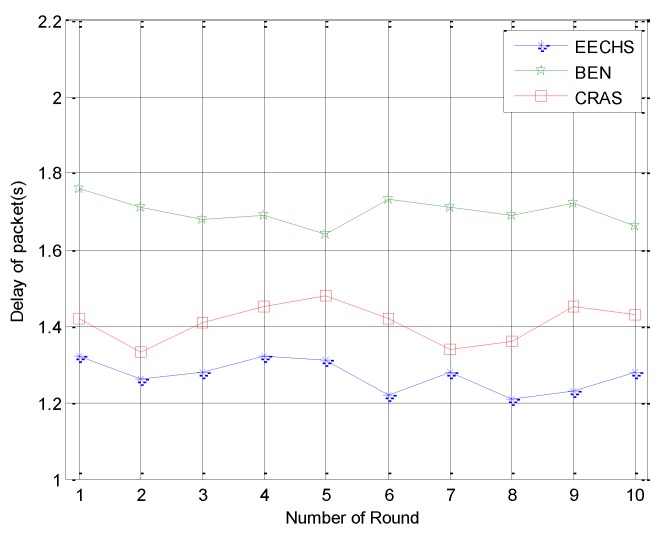
Experimental results for average packet delivery delay.

We next compared the average delay of packet delivery among these algorithms, and the results are shown in Figure 8. It can be observed that the proposed EECHS had the least delay among all the algorithms. This is due to the number of hops used by the different algorithms to transmit data. In both CRAS and BEN, the data is always transmitted to the CH and then to the sink. BEN always transmits data from the CH to the sink by multiple hops, while CRAS adopts the scheme of either multi-hop or one-hop routing. As for the proposed EECHS, for both CHs and CMs, as long as the rechargeable battery of the node is fully charged, the data is transmitted to sink directly. Thus, more data is transmitted to the sink directly in EECRH than in both CRAS and BEN. As a result, the EECRH had a better average packet delivery delay result than the other algorithms.

**Figure 9:**
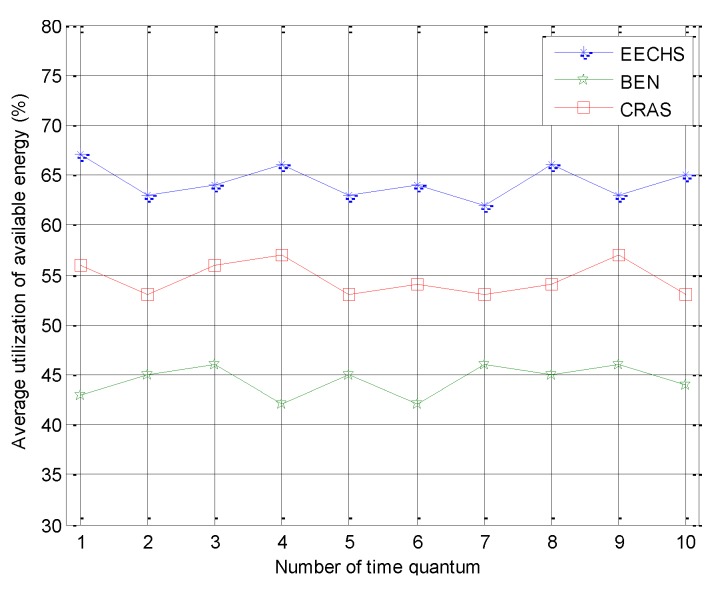
Experimental results for the average utilization of available energy from the environment.

Finally, we evaluated the average utilization of available energy from the environment among these algorithms. The results are shown in Figure 9, which indicates that the proposed EECHS had the best average utilization of available energy from the environment among all the compared algorithms. As discussed in Section 2, BEN is designed for traditional WSNs and it transmits data to the sink only by multi-hop routing and does not consider modifying the transmission radius of CHs and CMs according to the residual energies of their rechargeable batteries, which are changed dynamically according to the available energy from the environment. When more energy is available from the environment, BEN still adopts multi-hop routing for transmission. As a result, some energy from the environment is not used in BEN. In CRAS, although the uneven harvested energy among nodes is considered, the capacity limitation of the rechargeable battery is not considered, especially when the batteries of CMs are fully charged. Thus, a great deal of harvested energy is discarded. Compared to CRAS and BEN, the sensor node—both CH and CM—transmits data to the sink directly as long as its rechargeable battery is fully charged, and thus, it can harvest more energy from the environment than its competitors.

## 6. Conclusions

In this paper, we propose an energy-efficient CH selection algorithm called EECHS for EH-WSNs to select a reasonable node as CH and to take full advantage of the harvested energy. EECHS specifies a node as the SN for each cluster, which is used to monitor and store real-time information of residual energy for all CMs and the CH in the same cluster. According to the monitored result, the SN selects a corresponding CM as the new CH in each round to reduce the consumed energy caused by CH selection. Moreover, EECHS adjusts the transmission radius of some nodes if the corresponding batteries are fully charged. In order to evaluate EECHS, a series of simulation experiments were conducted. Simulation results verify that the proposed EECHS can provide effective performance for EH-WSNs and indeed had superior performance to its competitors.

The main limitation of this work is that it did not consider the relationship between the energy harvested by the sensor nodes from the environment and the energy consumed by the nodes to operate. If a node consumes more energy than it harvested in a given period of time, the node will still tend to perish. Therefore, in our future work, we intend to design another CH selection scheme by taking the relationship between the harvested and consumed energy into consideration. Furthermore, mobile devices such as unmanned aerial vehicles have become more and more popular and play an increasingly important role in our daily life. In our future work, we also want to include some mobile devices into the EH-WSNs to make the systems work more effectively.

## Figures and Tables

**Table 1:** Simulation parameter settings.

Parameter	Value
Size of network	500 m × 500 m
Number of sensor nodes	300
Location of the Sink	(505 m, 250 m)
$E_{ini}$	60 J
$E_{cap}$	100 J
$E_{ele}$	50 nJ/bit
$\epsilon_{fs}$	10 pJ/bit/m^2^
$\epsilon_{mf}$	0.0013 pJ/bit/m^4^
*T*	10
Size of each packet	200 bytes
α	1/3
β	10%
Size of solar panel	10 mm × 10 mm
The percent of sensor nodes in the shaded area	20%
